# Temperature effect in physicochemical and bioactive behavior of biogenic hydroxyapatite obtained from porcine bones

**DOI:** 10.1038/s41598-021-89776-2

**Published:** 2021-05-26

**Authors:** P. A. Forero-Sossa, J. D. Salazar-Martínez, A. L. Giraldo-Betancur, B. Segura-Giraldo, E. Restrepo-Parra

**Affiliations:** 1 Laboratorio de Física del Plasma, Universidad Nacional de Colombia- Manizales, Km 9 vía al aeropuerto, Campus La Nubia, Manizales, Colombia; 2 Centro de Investigación y de Estudios Avanzados del IPN, Lib. Norponiente 2000, Fracc. Real de Juriquilla, 76230 Querétaro, Qro México; 3 CONACYT-Centro de Investigación y de Estudios Avanzados del IPN, Lib. Norponiente 2000, Fracc. Real de Juriquilla, 76230 Querétaro, Qro México; 4 PCM Computational Applications, Universidad Nacional de Colombia - Sede Manizales, km. 9 vía al aeropuerto, Campus La Nubia, Manizales, Colombia

**Keywords:** Energy science and technology, Materials science

## Abstract

Biogenic hydroxyapatite (BHAp) is a widely used material in the biomedical area due to its similarities with the bone tissue mineral phase. Several works have been spotlighted on the thermal behavior of bone. However, little research has focused on determining the influence of calcination temperature in the physicochemical and bioactive properties of BHAp. In this work, a study of the physicochemical properties’ changes and bioactive response of BHAp produced from porcine femur bones using calcination temperatures between 900 to 1200 °C was conducted. The samples’ structural, morphological, and compositional changes were determined using XRD, SEM, and FTIR techniques. XRD results identified three temperature ranges, in which there are structural changes in BHAp samples and the presence of additional phases. Moreover, FTIR results corroborated that B-type substitution is promoted by increasing the heat treatment temperature. Likewise, samples were immersed in a simulated biological fluid (SBF), following the methodology described by Kokubo and using ISO 23317:2014 standard, for 3 and 7 days. FTIR and SEM results determined that the highest reaction velocity was reached for samples above 1000 °C, due to intensity increasing of phosphate and carbonate bands and bone-like apatite morphologies, compared to other temperatures evaluated.

## Introduction

During the last decades, the bioceramics field has generated particular interest due to the increasing demand to develop materials for dental and orthopedic applications. In this context, the hydroxyapatite (HAp) is one of the most widely used bioceramics materials in treating different diseases related to the musculoskeletal system because its composition is very similar to the mineral component of hard connective tissue^1^. Moreover, HAp exhibits bioactive behavior, biocompatibility, non-toxicity, and osteoconductive properties, necessary for healthcare applications^2^. HAp is usually used in its stoichiometric form (SHAp: Ca_10_(PO_4_)_6_(OH)_2_ and Ca/P = 1.67); nevertheless, trying to resemble its natural composition and inspired by it, its non-stoichiometric form can also be used, and commonly called BHAp^3^.

The BHAp formula can be represented by Ca_10−x_(PO_4_)_6−y_(OH)_2−(y+z)_^4^, where *x*, *y*, and *z* correspond to the different cations such as *Na*^+^, *Mg*^*2*+^, and *Sr*^*2*+^ and anions like *SiO*^*2*−^ and *CO*_*3*_^*2*−^, which can occupy calcium, phosphate or hydroxyl ions position in the lattice^5,6^. Any ion substitutions can modify the physical–chemical, biochemical, and physiological properties of the HAp and are feasible due to the HAp lattice flexibility and its structural stability^7^. According to Siddiqui et al.^8^, the *SiO*^*2*−^, *Mg*^*2*+^, and *CO*_*3*_^*2*−^ substitutions can enhance the rate of bone growth, bone remodeling and present a better resorption rate, respectively.

The *CO*_*3*_^*2*−^ substitutions are common in the natural bone, which can replace either hydroxyl (called A-type) or phosphate (called B-type)^8^. Likewise, it has been observed that the B-type substitution is related to a decrease in the a-axis and an increase in the c-axis in the HAp lattice. Besides, Siddiqui e*t al*.^8^ report that the formula is modified in this way Ca_10−x_(PO_4_)_6−x_(CO_3_)_x_(OH)_2−x_, where 0 < x < 2 for this type of substitution. It is believed that when BHAp is implanted, the native tissue growth toward the implant is stimulated, establishing a physical–chemical bond with the adjacent tissue. This behavior can improve its application in dental, maxillofacial, and implants devices^9–11^.

Animal bones (e.g., bovine, pig, goats, and chicken) are among the most common sources to prepare BHAp, using calcination or a combination of chemical and thermal treatments^4,12–15^. Each treatment promotes differences in BHAp properties like the degree of crystallinity, Ca/P ratio related to the solubility, ionic substitution, traces of impurities, and particle sizes or shapes, which impact the biological response^4,7,13,14,16^. The thermal process is usually involved during BHAp extraction from bones; hence, the temperature plays an important role in eliminating the organic and hazardous biological remnants and generating HAp lattice transformations.

The HAp lattice transformations, the phases, and properties of the BHAp obtained from animal sources are dependent on the temperature and heating and cooling rates and are also related to the trace minerals present in sources. Ellingham et al.^17^ describe that, depending on the heating rate, some of the calcium oxide (CaO), tricalcium phosphates (TCP’s), and tetracalcium phosphates (TTCP) thermal phases can be favored or not as the heating rate increases^17–20^.

Furthermore, the features of the BHAp can influence its response in a biological environment since some phases are more soluble than others, and the surface reactivity can enhance or harm its behavior. Bioactivity is the way to evaluate the capability of the biomaterial to form a bone-apatite layer. The velocity of layer formation is related to how bioactive the material is. It can be correlated with the BHAp source extraction, processing temperature, and ion substitution in the HAp lattice^21^. This kind of test is usually performed following the protocol established by Kokubo et al.^22^. Briefly, samples are immersed in an inorganic solution with an ionic concentration close to the human blood plasma, known as simulated body fluid (SBF), and the bone-like apatite layer formation as a function of the time is evaluated^22,23^.

Although the bioactive behavior of stoichiometric HAp and that of BHAp has been studied in different works, few studies have been found that present the evaluation of samples’ bioactive behavior, obtained from porcine bone as a function of the calcination temperature. Thus, this work aims to study the temperature treatment effect (from 900 to 1200 °C) on the BHAp samples in the bioactive response^24^. The bioactivity test was performed following Kokubo’s methodology and using ISO 23317: 2014 standard^25^. The structural and microstructural changes of the BHAp samples were followed using X-ray Diffraction (XRD), Fourier Transform Infrared Spectroscopy (FTIR), and Scanning Electron Microscopy (SEM) results.

## Experimental details

### Synthesis of BHAp powders

The BHAp powders were obtained according to the method reported elsewhere^24^. Briefly, the porcine bones were firstly hydrothermally treated to remove soft tissue and lipids. The samples were then exposed to microwave radiation at 700 W of power and rinsed using a ratio of 1:2 crushed bones/oxalic acid commercial-grade (PM 126, density 1.65 g/cm^3^, Merca Químicos y envases Caldas) to soften the peptides bonds. Later, bones were ground in a gravitational miller until the powder achieved particle sizes lower than 38 μm. The miller conditions used for this method were ball powder ratios 10:1 at 100 rpm. Finally, the resulting powders were subjected to heat treatment between 900 and 1200 °C, at a 5 °C/min heating rate and inertial cooling. The temperature was kept for 24 h to promote the HAp phase formation.

### Material tests and characterization

XRD was used to determine the phases and the crystalline quality of the calcium phosphates in the bone powders subjected to different thermal treatment temperatures. Samples were measured on a Rigaku MINIFLEX II equipment with CuK_α_ radiation (λ = 1.5406 Å) operating at 30 kV and 20 mA. Diffractograms were taken from 10° to 70° in a 2θ scale and using a resolution of 0.02°. The structural changes, the phase fraction, and the Ca/P ratio in each sample after thermal treatments were determined using Rietveld analysis of the XRD patterns by GSAS. The Ca/P ratio was calculated using Eq. (1), where Ca1, Ca2, and P are the occupancy values for those elements^20,26^.

1 $${\text{Ca/P}} = \left[ {4*{\text{Oc}}\left( {{\text{Ca1}}} \right) \, + \, 6*{\text{Oc}}\left( {{\text{Ca2}}} \right)} \right]/\left[ {6*{\text{Oc}}\left( {\text{P}} \right)} \right]$$

The functional groups associated with calcium phosphate were identified using the FTIR technique. The spectra were acquired between 400 to 4000 cm^−1^ with a resolution of 4 cm^−1^ and 32 scans on a Bruker Alpha equipment equipped with ATR platinum Diamond 1 accessory. The morphological evaluation was performed using SEM micrographs acquired on a FEI QUANTA 250 scanning electron microscope. All micrographs were collected using 12.5 kV electron acceleration voltage. Before measurement, samples were coated with a gold–palladium thin film to make them conductive.

The materials were evaluated in SBF following the methodology described by Kokubo et al.^22^. Powders were compacted into pellets of 13 mm in diameter, using a uniaxial press at 100 MPa to perform the bioactive assay. For these samples, no additional thermal or roughness treatments were applied. These pellets were immersed in SBF for 3 and 7 days, using an area/volume ratio of 0.76 mm^2^/mL. The structural and morphological changes as a function of the soaking time after the bioactivity test were analyzed through FTIR and SEM, using the same conditions mentioned before.

## Results

**Figure 1:**
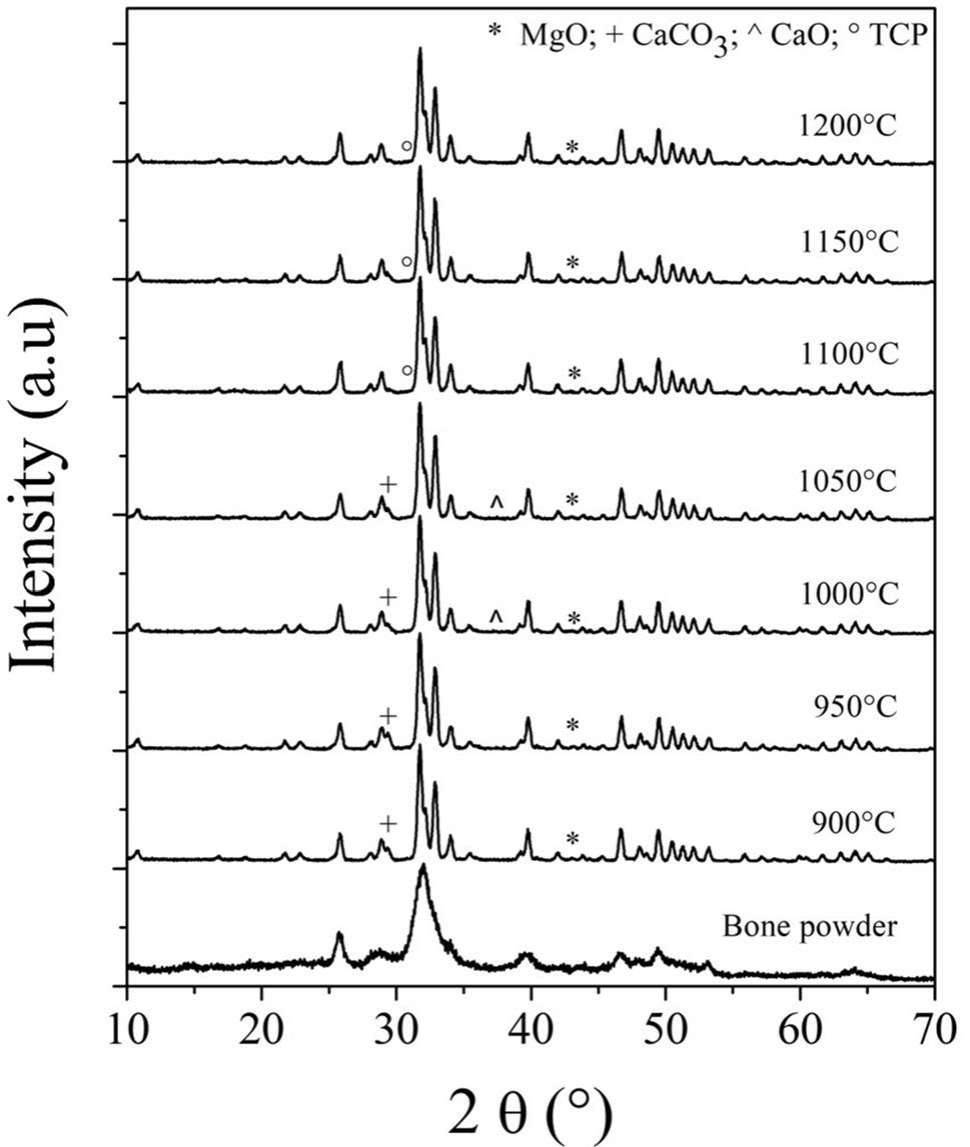
XRD patterns of the samples treated at different calcination temperatures.

The structural changes of the bone powder and the BHAp’s samples at different thermal treatments were evaluated using X-ray diffraction patterns shown in Fig. 1. HAp (PDF # 09-0432) was identified as the main phase and MgO (PDF # 45-0946) as the second phase^14^. However, additional phases were observed as a function of the temperature used in the treatment. Initially, in samples obtained between 900 and 950 °C, a peak corresponding to the calcite (CaCO_3_) phase (PDF # 47-1743) was found at 29.40° in 2θ^27,28^. In samples thermally treated between 1000 and 1050 °C, a small contribution at 37.48° was found associated with the calcium oxide (CaO) phase (PDF # 37-1497), in addition to the calcite peak^29^. Finally, the samples treated at temperatures higher than 1100 °C present α-TCP (PDF # 29-0359) and β-TCP (PDF # 09-0169) as additional phases^30,31^ . The weight percentages of the phases found in the samples, the changes in the volume of the HAp cell, and the Ca/P ratio calculated using the atoms’ occupancies in the cell obtained by Rietveld refinement through Eq. (1) are summarized in Table 1.

**Table 1:** Structural parameters obtained for the samples from XRD patterns by Rietveld refinement.

Thermal treatment (°C)	Phase wt. (%)						Volume (Å^3^)*	Ca/P
	HAp	MgO	CaCO_3_	CaO	α-TCP	β-TCP		
900	93.233	0.750	6.017	–	–	–	529.974	1.656
950	91.221	0.798	7.982	–	–	–	528.415	1.589
1000	95.653	0.188	3.871	0.288	–	–	529.896	1.704
1050	92.529	1.185	6.090	0.196	–	–	527.827	1.530
1100	96.461	0.305	–	–	1.926	1.308	529.934	1.700
1150	88.914	1.992	–	–	1.700	7.394	527.907	1.672
1200	90.363	0.475	–	–	2.505	6.658	529.426	1.694

*Stoichimetry cell volume = 527.52 Å^3^.

**Figure 2:**
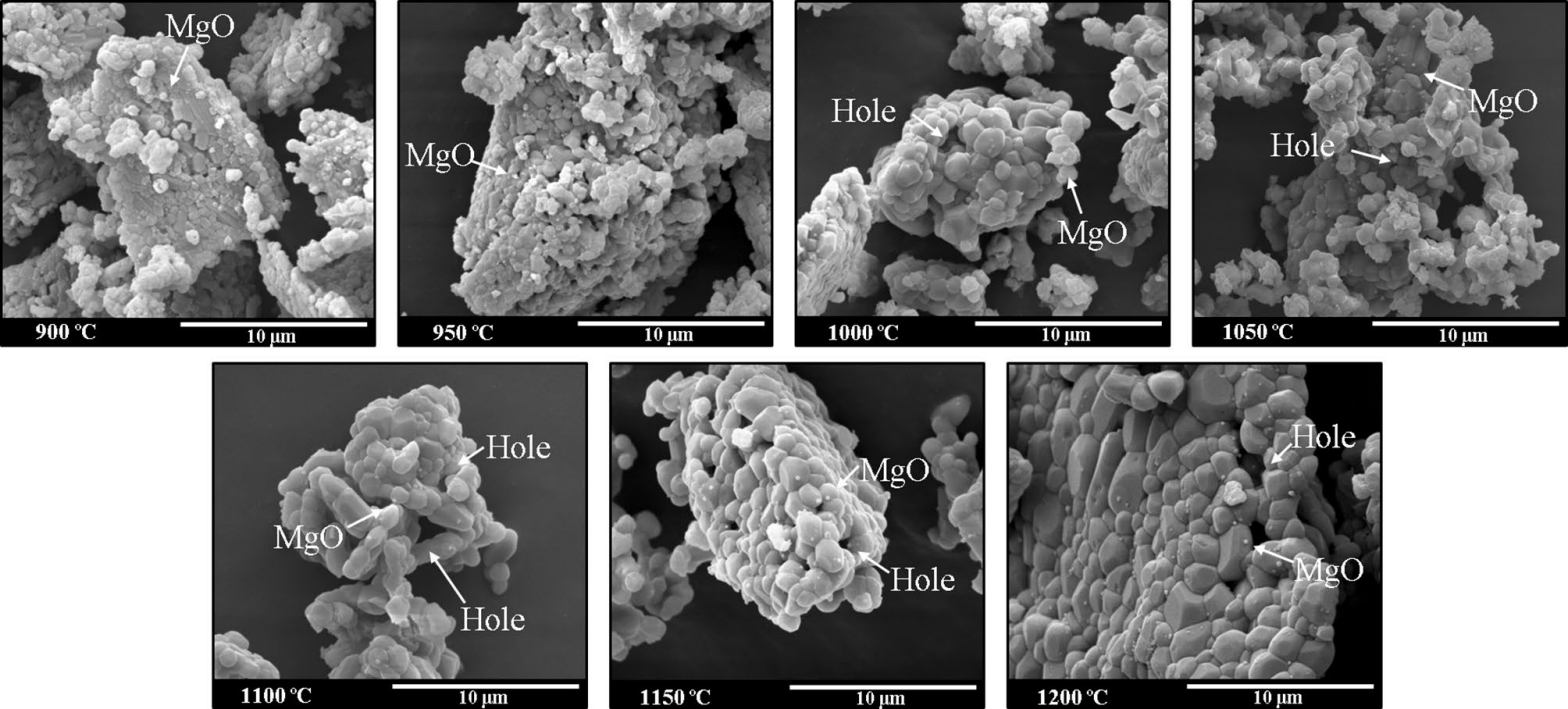
SEM images of the samples thermally treated at different calcination temperatures.

The morphological changes of the BHAp samples obtained as a function of the treatment temperature were studied using the SEM micrographs and are shown in Fig. 2. Grains ranging from a few hundred nanometers to a few microns are observed in the powders treated with a temperature of 900 and 950 °C. Additionally, brick-shaped grains with a few microns and little coalescence between them are observed due to the low calcination temperature. On the other hand, a specific intergranular porosity is observed. In the samples treated using temperatures higher than 1000 °C, both the grains’ growth generating micrometric sizes particles and voids’ appearance are observed^18^. Finally, in the images of all the samples, sub-micrometric spherical grains are seen, which can be associated with the magnesium oxide phase identified by XRD, taking into account what was reported by Ramirez-Gutierrez et al.^14^.

**Figure 3:**
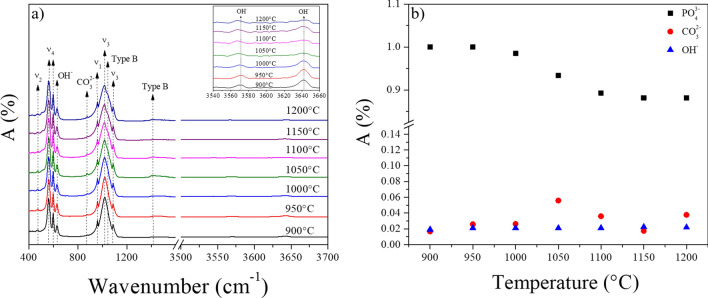
(**a**) FT-IR spectra of the samples treated at different calcination temperatures. (**b**) Intensities of the normalized functional groups as a function of the calcination temperature.

Figure 3 (a) shows the FTIR spectra and (b) the variation of the functional group intensity associated with BHAp for the samples obtained at different calcination temperatures. From the FTIR spectra, in Fig. 3a, the phosphate groups characteristic bands were identified at 961 cm^−1^ (υ_1_), 462 cm^−1^ (υ_2_), 1021 and 1087 cm^−1^ (υ_3_), 561 and 598 cm^−1^ (υ_4_)^33–35^. The bands at 1044 and 1415 cm^−1^ were also identified, corresponding to the incorporation of *CO*_*3*_^*2*−^ in the *PO*_*4*_^*3*−^ sites in the HAp cell^36–38^. This change is typical of the B-type substitution in the lattice. Particularly, the shoulder at 1044 cm^−1^ is not observed in the samples with treatments of 900 and 950 °C, which may be associated with a lower B-type substitution in the HAp cell for these calcination temperatures. This behavior will be reviewed in the discussion section^8,36,39^. Additionally, the bands at 629 and 3570 cm^−1^, corresponding to the hydroxyl group were also identified; these bands are characteristic of the HAp phase^40^. Finally, Fig. 3b shows the changes in the functional groups’ intensities for phosphate υ_3_ at 1021 cm^−1^, carbonate at 1415 cm^−1^, and hydroxyl at 3570 cm^−1^ as a function of the calcination temperature. In particular, the phosphate group’s intensity for the range of temperatures from 1050 to 1200 °C decreases with respect to the other calcination temperatures^19^. On the other hand, the functional groups’ intensities of the carbonate and hydroxyl ions do not significantly change in the range of studied temperatures^19,41^.

**Figure 4:**
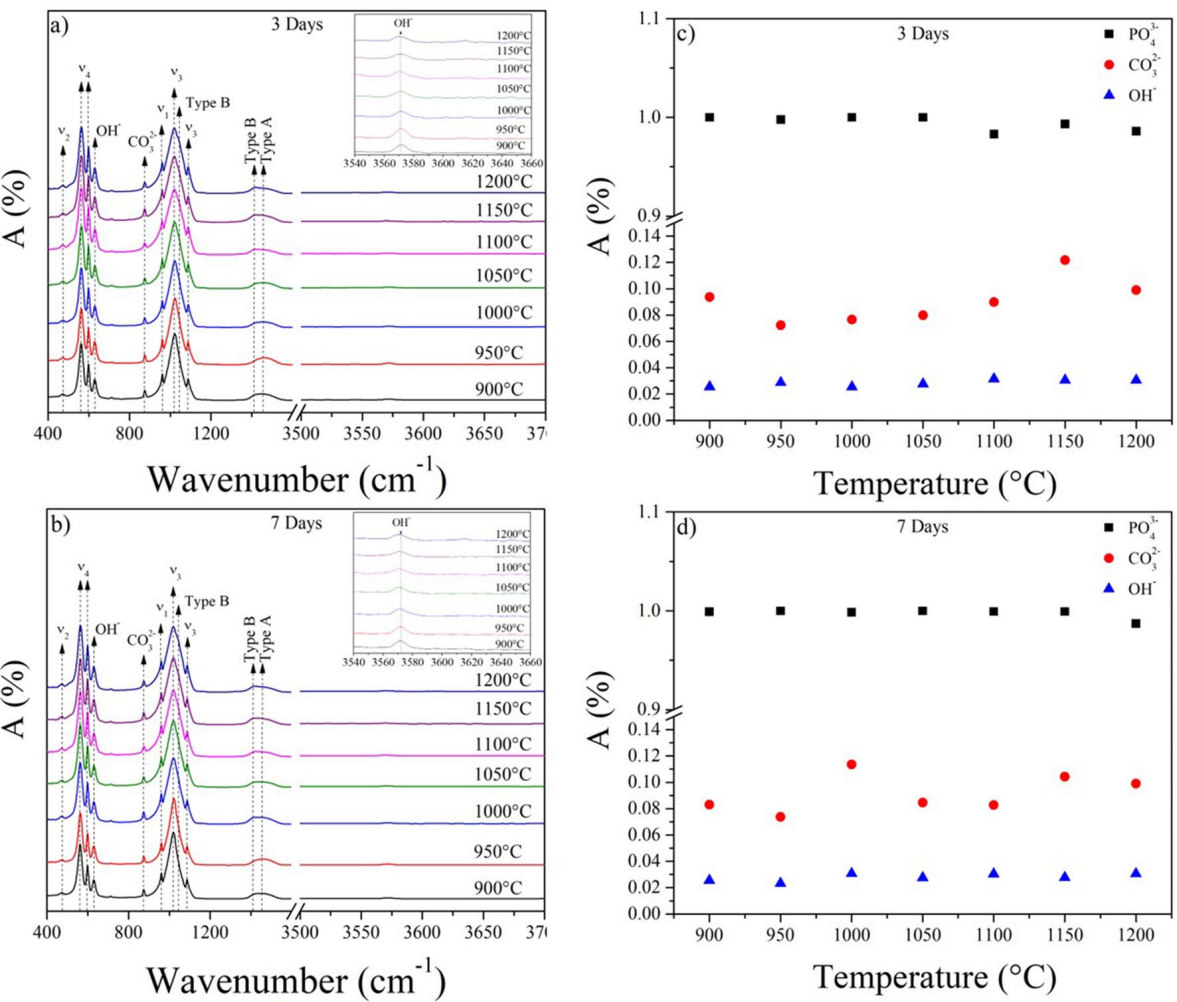
FT-IR spectra of samples after (**a**) 3 days and (**b**) 7 days soaking time in SBF Intensities of the normalized functional groups for samples after (**c**) 3 days and (**d**) 7 days of soaking time in SBF.

Figure 4 presents the FTIR spectra and the functional groups’ intensity for the samples obtained with different temperatures after 3 and 7 days of immersion in SBF. In Fig. 4a,b, the same functional groups identified in Fig. 3a were observed. However, in the range between 1400 to 1600 cm^−1^ the vibrations at 1411 and 1456 cm^−1^ associated with the B- and A-type substitution were identified. Besides, the contribution at 1415 cm^−1^ increases its intensity compared to the samples before SBF exposure. Likewise, the shoulder at 1044 cm^−1^ is not observed in the samples treated at 900 and 950 °C, regardless of the immersion time. In Fig. 4c,d the intensities of the phosphate and carbonate functional groups increase with respect to the samples that were not exposed in SBF (from Fig. 3b). Furthermore, for samples with calcination treatments above 1050 °C, the intensity of the vibrational mode υ_3_ of the phosphate group shows a slight increase with increasing immersion time in SBF^36,42^.

**Figure 5:**
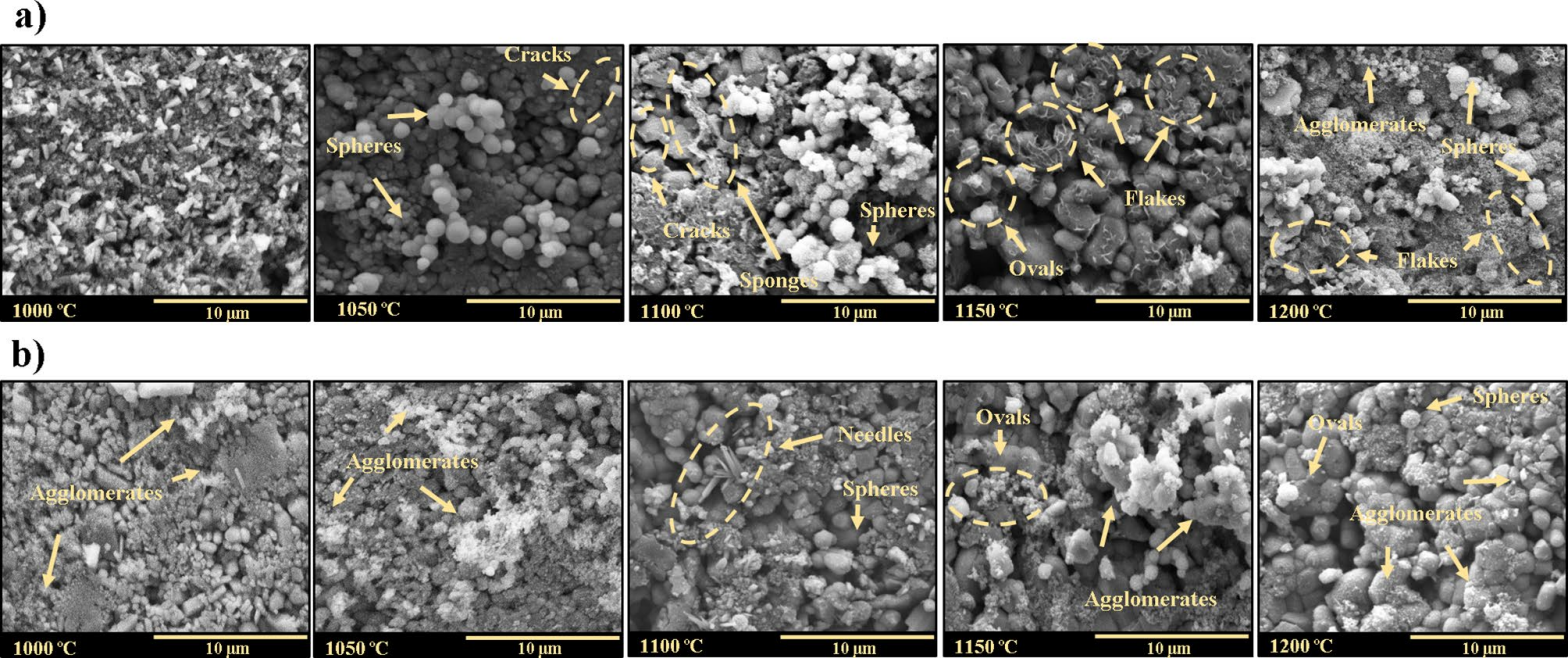
SEM images of the samples treated at different calcination temperatures and after soaking in SBF during (**a**) 3 days and (**b**) 7 days.

Moreover, samples thermally treated at 900 and 950 °C do not present the surface’s apatite layer’s formation after the soaking time in SBF based on FTIR and SEM results. Figure 5 shows morphological changes of samples as a function of the treatment temperature (from 1000 to 1200 °C) and the SBF immersion time. For the micrographs of the samples treated at 1000 °C, after 3 days of immersion in SBF (Fig. 5a), pyramidal and brick-like morphologies are observed, which are not typical of bone-like apatite. However, after 7 days of immersion, it is possible to observe agglomerates with cauliflower-like morphology that is associated with the formation of apatite on the surface (Fig. 5b)^43^. Particularly, in the samples treated at 1050 °C after 3 days of immersion, cracks on the material’s surface and agglomerated spheres of micrometer size are observed (Fig. 5a). After 7 days of soaking, cauliflower-like morphologies are observed (Fig. 5b), which according to the literature, are typical formations of apatite growth on the surface of the material^43,44^. Regarding the samples treated at 1100 °C after 3 days of immersion, two types of morphologies are observed: spheres- and sponge-like, and the presence of cracks (Fig. 5a). Similarly, at 7 days, the formation of needles and spheres is observed on the ceramic surface (Fig. 5b)^43^. For the samples treated at 1150 °C with 3 days of soaking, oval and flake-like morphologies are observed with micro- and sub-micrometric sizes, respectively. The flakes are interconnected and grown into oval grains (Fig. 5a). At 7 days of exposure, the oval grains were observed; likewise, the formation of agglomerated sub-micrometric spheres is evidenced (Fig. 5b)^45^. Finally, for the samples treated at 1200 °C after 3 days of exposure, agglomerates of sub-micrometric spheres, interconnected flakes, and micrometric spheres are observed (Fig. 5a). Then, for the 7 days of immersion, the presence of oval and spherical grains and the formation of sub-micrometric spherical agglomerates is evident on the surface (Fig. 5b)^46,47^.

## Discussion

The behavior of bone powder without heat treatment, observed in the diffraction patterns in Fig. 1, is associated with the nanometric size of the bone crystals, as well as its organic component. The nanometric size and organic component generate peaks with poorly defined widths in the XRD patterns, previously reported by Giraldo-Betancur et al.^4^ and Londoño-Restrepo et al.^18^. Additionally, in Fig. 1 and Table 1, the changes that the bone powder undergoes can be observed when subjected to different calcination temperatures and how each of them can favor the appearance of additional phases. The MgO phase’s presence is associated with the release of *Mg*^*2*+^ from the BHAp cell and its subsequent reaction with oxygen in the atmosphere. This phenomenon occurs at temperatures above 600 °C, as was reported by Ramirez-Gutierrez et al.^14^. The calcination temperature promotes the release of *Mg*^*2*+^ ions from the cell, as already mentioned. Then, the reaction between the *Mg*^*2*+^ and the *O*^−*2*^ of the atmosphere occurs to form MgO (△H_f_ = − 601 kJ/mol), whose enthalpy of formation is favorable compared to Mg(OH)_2_ (△H_f_ = − 924.7 kJ/mol), which would be the other compound that could be formed. Hence, MgO is the most thermodynamically stable compound^48^. Likewise, the presence of CaCO_3_ in the treated samples in the range of 900 to 950 °C is attributed to the biogenic source of HAp^49,50^, since this range of heat treatment temperature is not sufficient to promote the dissociation of CaCO_3_ into CaO and CO_2_ yet^51^.

In the samples treated above 1000 °C, the CaCO_3_ and CaO phases were identified. The total decomposition of calcite depends on the working atmosphere and can occur above 900 °C, where CaO is obtained, as reported by Galán et al.^51^. Subsequently, in the samples obtained at temperatures in the range of 1100 to 1200 °C, CaO and CaCO_3_ presence was not evidenced. However, the α- and β-TCP phases were identified. The CaO and CaCO_3_ phases’ disappearance, together with the formation of tricalcium phosphates, could be related to a possible reaction of CaCO_3_ with the phosphate ions released from the HAp lattice due to the *CO*_*3*_^*2*−^ incorporation. The incorporation of carbonate ions into the HAp lattice would lead to the release of phosphate ions from the cell, resulting in the formula Ca_10−x_(PO_4_)_6−x_(CO_3_)_x_(OH)_2−x_ reported by Siddiqui et al.^8^. Likewise, it has also been reported that tricalcium phosphates can be obtained from the reaction between calcium carbonate and phosphate ions at temperatures around 1150 °C, according to Chabchoub et al. and Bohner et al.^52–54^. Furthermore, the lattice in the BHAp samples is expanded, compared to the stoichiometric one, according to the cell volumes’ values obtained and reported in Table 1. The Ca/P ratio differs from the stoichiometric HAp value for all calcination temperatures (Table 1). This behavior is due to the substitutions presented by BHAp related to its biogenic source^8,55^.

The morphologies for samples prepared at temperatures of 900 and 950 °C observed in Fig. 2 are associated with the loss of organic compound. Likewise, the particles’ coalescence is also due to the calcination temperature. According to what was reported by Sofronia et al. and Londoño-Restrepo et al.^3,40^, the coalescence effect occurs in this range of temperature for calcined bovine bone powders. Additionally, Londoño-Restrepo et al.^18^ reported coalescence phenomena in the porcine bone powders associated with crystal growth due to the calcination temperature. They also mentioned that, as the calcination temperature increases, the crystal growth continues, and it is observed on the c-axis.

In this work, the growth of grains occurs in the c-axis in the samples treated at temperatures higher than 1000 °C, which agrees with previous reports for the growth of HAp crystals^56,57^. This growth generates intergranular voids of greater size than those observed in the samples’ images obtained with lower temperatures. Finally, the images show sub-micrometric spherical grains associated with the MgO phase observed in XRD. Ramirez-Gutierrez et al.^14^ and Forero-Sossa et al.^20^ have also reported these formations in previous works.

Regarding the functional groups’ behavior obtained by FTIR for the range of 900 and 950 °C, some authors such as Sofronia et al.^40^ and Asadollahzadeh et al.^44^ mentioned the reduction of the intensities of the carbonate groups at these calcination temperatures. However, these authors did not carry out thermal treatments above this temperature. In particular, Sroka et al.^36^ studied the influence of the carbonate ions in HAp, finding that when this ion enters the cell and replaces the phosphate ion, the shoulder’s presence at 1044 cm^−1^ is observed in the FTIR spectra using the ATR mode^36^. In this work, this shoulder is observed for samples with heat treatment above 1050 °C. Likewise, a decrease in the phosphate group’s intensity was observed, which could be associated with incorporating the carbonate ion for these samples in this temperature range.

Furthermore, based on the SEM and FTIR results, the non-formation of bone-like apatite in the samples at 900 and 950 °C could be associated with no carbonate substitution in the HAp cell at these temperatures^8^. On the other hand, Kokubo’s solution in a static environment has been widely criticized due to its low reaction rate compared to other simulated biological solutions. Using these characteristics is possible to obtain false negatives, such as the β-TCP phase, since this does not show bone-like apatite growth on the surface when immersed in SBF. However, it is a biomaterial known for its biological response in in-vivo assays^54,58^. Based on this, it is possible that in other simulated media, such as Hank’s solution, the formation of different phases can be observed due to the ionic interaction between the ceramic and the fluid, as mentioned by Forero-Sossa et al.^20^.

Moreover, the samples with calcination temperatures above 1000 °C showed significant changes in the functional groups’ intensities and the samples’ surface morphology. These results determined that bone-like apatite forms on the samples’ surface were obtained in the temperature range of 1000 to 1200 °C when exposed to SBF in this temperature range. Particularly, the samples treated with temperatures higher than 1100 °C show the growth of morphologies associated with bone-like apatite. This group of samples’ higher bioactivity could be related to incorporating the carbonate ion in the HAp lattice and the combination of phases (HAp + TCP). According to various works, the phase combination presents improved bioactivity compared to individual ones^59–61^. Some authors have also reported that the solubility of TCP in physiological environments is greater than that of HAp, which would increase the reaction speed of samples that present these phases. Those results agree with the findings observed in this work^32,54^.

## Conclusions

The effect of the calcination temperature on the physicochemical properties and the bioactive response of BHAp from the porcine source was determined. It was found that the HAp and MgO phases are present in all the samples with the different calcination temperatures studied. Additionally, between 900 and 950 °C, the CaCO_3_ phase was found. Likewise, between 1000 and 1050 °C, the CaO phase and CaCO_3_ coexist. Finally, , between 1100 and 1200 °C, the formation of TCP’s was identified. This result could be associated with the reaction between CaCO_3_ and phosphate ions released from the HAp cell due to the carbonate ions incorporation on it. High calcination temperatures mediate the proposed mechanism.

Using the FTIR results, it was determined that the replacement of the phosphate ions by carbonate ones is greater in the samples prepared at high temperatures. Then, the B-type substitution is promoted at calcination temperatures between 1150 and 1200 °C, likewise, the A- and B-type substitutions were favored after the bioactivity test.

The samples thermally treated at 900 and 950 °C did not show changes in the spectra and the micrographs during the bioactivity test. However, for the samples treated at temperatures above 1000 °C, there were changes related to bone-like apatite formation on the samples’ surface. Finally, for the samples with a calcination temperature between 1100 and 1200 °C, tricalcium phosphates’ presence generates a higher reaction speed than the other samples that did not present these additional phases.

## Supplementary Information

Supplementary Information.
