# Supplementary material for: Temperature effect in physicochemical and bioactive behavior of biogenic hydroxyapatite obtained from porcine bones
Source: Sci Rep. 2021 May 26;11:11069. doi: 10.1038/s41598-021-89776-2 (PMC8154992; doi:10.1038/s41598-021-89776-2)
Supplement: Supplementary file 1 — Supplementary Information. [file 41598_2021_89776_MOESM1_ESM.pptx]

## Slide 1
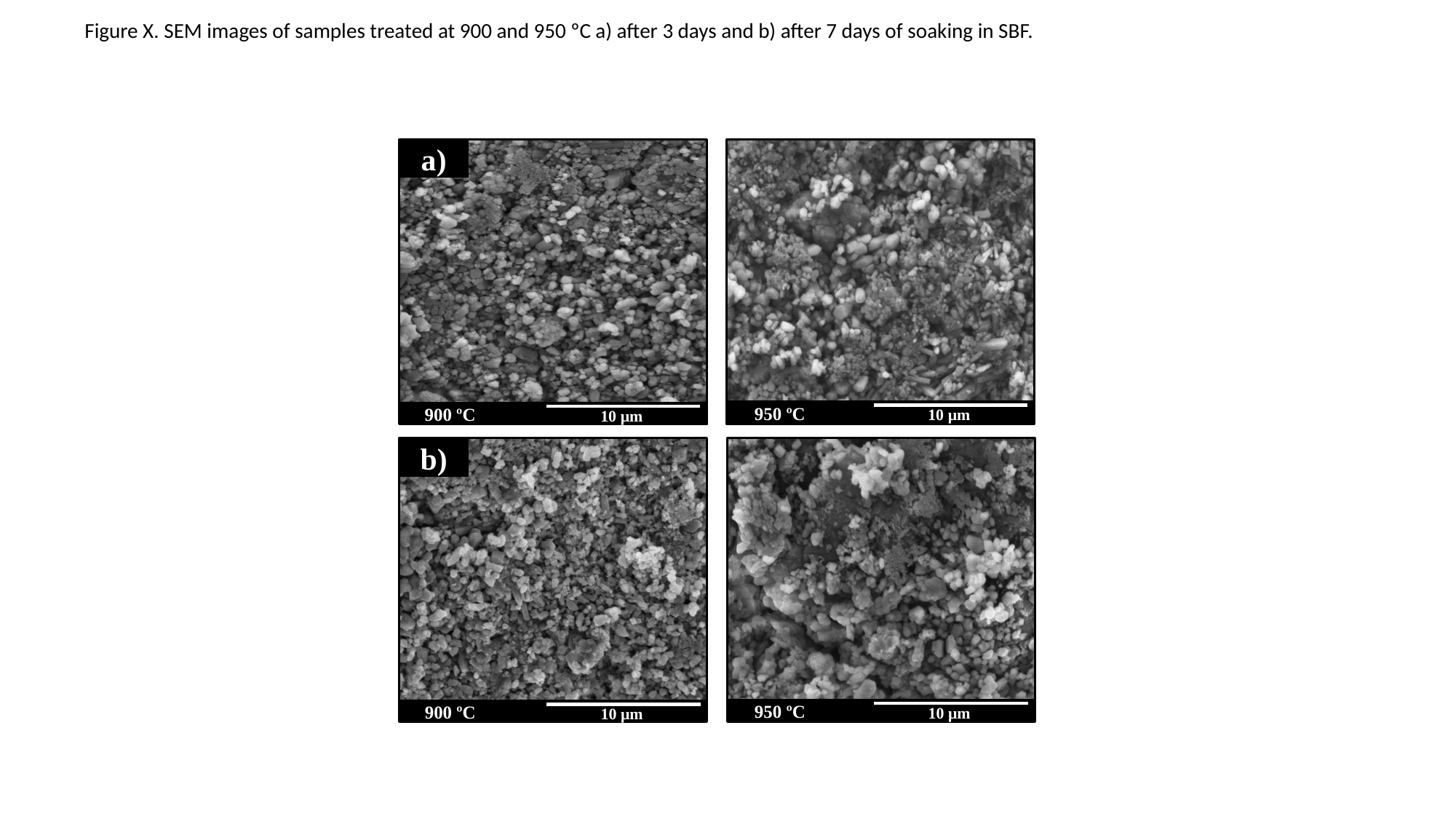

Figure X. SEM images of samples treated at 900 and 950 ºC a) after 3 days and b) after 7 days of soaking in SBF.
a)
950 ºC
900 ºC
10 μm
10 μm
b)
950 ºC
900 ºC
10 μm
10 μm
